# Pattern of neurological admissions in the tropics: Experience at Kano, Northwestern Nigeria

**DOI:** 10.4103/0972-2327.70875

**Published:** 2010

**Authors:** L. F. Owolabi, M. Y. Shehu, M. N. Shehu, J. Fadare

**Affiliations:** Department of Medicine, Aminu Kano Teaching Hospital/Bayero University Kano, Nigeria

**Keywords:** Kano, Nigeria, neurologic disease

## Abstract

**Background:**

Kano is the most populated state in Nigeria with a population totaling 9,383,682. The pattern of neurologic diseases in this area is not known.

**Objective:**

To determine the of pattern of neurologic diseases warranting admission in a tertiary hospital in Kano and compare it with those elsewhere in the country with the view to using the data generated as a baseline for planning purposes and for future studies.

**Materials and Methods:**

The medical records of all cases admitted with neurologic diseases in the Aminu Kano Teaching Hospital, Kano between January 2005 and September 2008, were retrospectively reviewed and the frequency of neurologic diseases, sex, age, and outcome of these diseases analyzed.

**Result:**

Stroke, predominantly ischemic, accounted for 77.6% of the neurological cases for the period of study. Central nervous system infections, comprising mainly of meningitis and tetanus, accounted for 6.6% (64) and 3% (29) of cases, respectively. The myelopathies were the cause of neurologic admissions in 5.4% (53) with paraplegia and quadriplegia resulting from myelopathies accounting for 5% (49) and 0.4% (4) of the cases. Hypertensive encephalopathy and status epilepticus as the causes of admissions accounted for 1.6% each. Gullain Barre syndrome, Parkinson’s disease, and cerebral malaria were relatively rare causes of neurologic admissions in this study. The average duration of hospitalization was 25 days, and regarding outcome, 219 (22.4%) of these cases died.

**Conclusions:**

Stroke appeared to be the most common neurologic admission and the most common cause of neurologic and medical death in Kano as observed in other regions of the country and a little over one-fifths of stroke patients die. Central nervous system infections mainly meningitis and tetanus are the next common cause of admission. In view of these findings, the provision of a regional stroke unit, the improvement of the sanitary conditions of the home and environment; the widespread use of immunizations against meningitis, tetanus cannot be over-emphasized. These interventions will go a long way to reduce morbidity and mortality of stroke and neurologic infections.

## Introduction

Kano is a state located in the North-Western Nigeria, it extends over an area of about 42,592.8 sq kilometers with a population density of about 466/km.^2^ The state borders Katsina state to the north-west, Jigawa state to the North-east, and Bauchi and Kaduna to the south. It is the most populated state in Nigeria with a population totaling 9,383,682.[1] It is home to 44 local government areas. The predominant occupations in the area are trading and farming. This study was conducted in the only teaching hospital in the state; the department of medicine of this institution has 54 bed wards (36 males, 18 females); the hospital has neurodiagnostic facilities like CT scan, EEG, EMG, and nerve conduction test machines.

The hospital has a wide catchment area, particularly for neurologic conditions, including the neighboring states. However, the pattern of neurologic admissions in this area is not known. Therefore, this study was undertaken to determine the pattern of neurologic diseases warranting admission in a tertiary hospital in Kano and compare it with those elsewhere in the country with the view to using the data generated as a baseline for planning purposes and for future studies.

## Materials and Methods

The records of all medical admissions, between January 2005 and July 2007, were collected from the medical wards and the medical records department of the hospital. Patients above 12 years of age were included in the study. Figures of hospital admissions and deaths during the same period were also collected from the medical records department. The identified cases were then classified and only cases confirmed as neurological were further analyzed. The data extracted from the patients’ records included age, sex, date of admission, diagnosis, date of discharge, and outcome (whether discharged, died, discharged against medical advice or absconded, or referred to other tertiary centers or departments). These data were then analyzed using SPSS version 15. The neurologic diseases were grouped into the following diseases: stroke, TIAs (transient ischemic attacks), spinal cord diseases (included Pott’s disease, cervical and lumbar spondylosis, and disc disease), tetanus, Parkinson’s disease and other movement disorders, epilepsies, cerebellar syndromes, meningitis (bacterial, tuberculous and viral), encephalitis, primary CNS tumors, and neuropathies.

The diagnoses were made based on clinical evidence and laboratory confirmation in the majority of the patients. The laboratory investigations, depending on the suspected neurologic disease process, included Full blood count, ESR, ECG, X-rays, serological tests serum biochemistry, EEG CSF analysis, microbiology, and histopathology. Not all the patients who required neuroimaging had it (CT scanning or MRI) because of financial constraints. Carotid angiography, viral studies, and other sophisticated neurologic investigative modalities were not utilized in these diagnoses because of unavailability of these facilities.

The diagnosis of Stroke was made in the presence of sudden onset focal or global neurological deficit lasting more than 24 hours or resulting in death for which there was no apparent cause but cerebrovascular. Categorization of stroke into ischemic or hemorrhagic stroke was done in accordance with WHO stroke criteria, and or Brain CT scan. Diagnosis of meningitis was made in the presence of headache, stiff neck, altered mental status, white blood cells in spinal fluid with or without isolation of *pathogens* from cerebrospinal fluid (CSF), tetanus was diagnosed in the presence of acute onset of hypertonia and/or painful muscular contractions (usually of the muscles of the jaw and neck), and generalized muscle spasms without other apparent medical cause.[2] Parkinsonism was diagnosed in the presence of three out of tremor, rigidity, akinesia or bradykinesia, and postural instability. Diagnosis of Gullain Barre syndrome was in accordance with Asbury clinical and laboratory criteria.[3]

## Results

A total of 980 patients were admitted with neurologic disorders during the period under review. The frequency of neurologic diagnosis, sex, age, duration of admission, and outcome of the neurologic diseases were analyzed. Neurologic diseases accounted for 980 of 6282 (15.6%) medical admissions in the hospital during the period. The sex distribution of patients was 586 (60%) males and 394 (40 %) females [Table 1], giving a sex ratio (M: F) of 3:2. The ages of the patients ranged from 12 to 90 years with a mean of 53.2 years [Table 2]. Stroke, predominantly ischemic, accounted for 77.6% of the neurological cases for the period of study. Central nervous system infections, comprising mainly of meningitis and tetanus, accounted for 6.6% (64) and 3% (29) of cases, respectively. The myelopathies were the cause of neurologic admissions in 5.4% (53) with paraplegia and quadriplegia resulting from myelopathies accounting for 5% (49) and 0.4% (4) of the cases, respectively. Hypertensive encephalopathy and status epilepticus as the causes of admissions accounted for 1.6 % each. Gullain Barre syndrome, Parkinson’s disease, and cerebral malaria were relatively rare causes of neurologic admissions in this study [Table 1]. The shortest duration of stay in the hospital was 24 hours and the longest duration of hospitalization was 96 days with a mean of 25 days. Regarding outcome, 753 (76.8%) of the patients, including those referred for neurosurgical intervention, were discharged, 219 (22.4%) died, and 8 (0.8%) were discharged against medical advice (DAMA) or absconded [Table 3].

**Table 1:** Distribution of neurologic diagnosis

Neurological disorders	Age range (Yrs)	Frequency			Percentage
		M	F	Total	
Stroke	20-90	456	304	760	77.6
Meningitis	14-44	30	34	64	6.6
Tetanus	18-50	23	6	29	3.0
Myelopathy (paraplegia)	22-60	28	21	49	5.0
Hypertensive encephalopathy	38-86	14	2	16	1.6
Status epilepticus	45-56	4	12	16	1.6
Parkinsonism	50-68	4	0	4	0.4
GB syndrome	15-35	5	1	6	0.6
Myelopathy (qadriplegia)	46-58	3	1	4	0.4
Cerebral tumor	52-65	9	9	18	1.8
Cerebral malaria	12-22	4	2	6	0.6
Snake bite	42-55	2	0	2	0.2
Encephalitis	15-21	2	2	4	0.4
Cerebral abscess	36-40	2	0	2	0.2
Total		586	394	980	100

**Table 2:** Distribution of age range of the patients

Age range	Frequency	Percentage
12-20	36	3.7
21-30	66	6.7
31-40	90	9.2
41-50	140	14.3
51-60	400	40.8
61-70	198	20.2
71-80	30	3.1
81-90	20	2.0
Total	980	100

**Table 3:** Distribution of outcome of the cases

Neurolological disorders	Outcome frequency / (Percentage of individual cases)			Total	Percentage
	Survived	Died	DAMA*		
Stroke	580 (76.3)	175 (23)	5 (0.7%)	760	77.6
Meningitis	46 (71.9)	17 (26.6)	1 (1.5%)	64	6.6
Tetanus	19 (65.5)	10 (34.5)	0	29	3.0
Myelopathy (paraplegia)	40 (81.6)	7 (14.3)	2 (4.1%)	49	5.0
Hypertensive encephalopathy	15 (93.8)	1 (6.2)	0	16	1.6
Status epilepticus	14 (87.5)	2 (12.5)	0	16	1.6
Parkinsonism	4 (100)	0	0	4	0.4
GBS	4 (66.7)	2 (33.3)	0	6	0.6
Myelopathy (quadriplegia)	3 (75)	1 (25)	0	4	0.4
Cerebral tumor	16** (88.9)	2 (11.1)	0	18	1.8
Cerebral malaria	5 (83.3)	1 (16.7)	0	6	0.6
Snake bite (with neurologic manifestat)	1 (50)	1 (50)	0	2	0.2
Encephalitis	4 (100)	0	0	4	0.4
Cerebral abscess	2** (100)	0	0	2	0.2
Total	753	219	8	980	100

* Discharged against medical advice;

** Referred for neurosurgical intervention, Figures in parenthesis are in percentage

## Discussion

This study showed that neurologic disorders are common in Kano. These diseases included stroke, meningitis (mainly bacterial and tuberculous), spinal cord disorders (mainly tuberculous spondylitis, cervical and lumbar spondylosis,), tetanus, hypertensive encephalopathy, status epilepticus of due to various causes, Parkinson’s disease, primary CNS tumors, and acute inflammatory polyneuropathy. These diseases constituted 15.6% of medical admissions. Generally, there was no significant sex predilection in these diseases. The mean age of the patients was 53.2 years. The highest peak age range of cases was between 51 years to 60 years. The major causes of admission in the Parkinson’s disease patients were sepsis from urinary tract infections and aspiration pneumonitis. In terms of the frequency of occurrence, stroke was the most common. Central nervous system infections (mainly meningitis and tetanus), with most of the cases of bacterial meningitis occurring during meningococcal meningitis epidemics as the study site is located within meningitis belt of Africa. Spinal cord diseases and hypertensive encephalopathy were also among the most common neurological disorders admitted. These four major disorders constituted 92.2% of all neurologic cases admitted.

Hypertension was the most common risk factor for stroke as it was the case in 97.5% cases. Tuberculosis of the spine was the predominant cause of spinal cord diseases (60%). Other less common neurologic diseases were Parkinson ’s disease, movement disorders (2.2%) and neuropathies (1.8%).

These findings are similar to those of Talabi in a similar study conducted in University College Hospital, Ibadan (South western Nigeria) who reported frequencies of: stroke (50.4%), tetanus (14.2%), meningitis (12.4%), and myelopathies (8.1%). Stroke, though in smaller proportion, was also reported as the most common neurologic diseases in his study.[4] In a similar study by Ojinni and Danesi in Lagos (Southern Nigeria), neurological admissions accounted for 19.6 of total medical admissions. Cerebrovascular diseases were the most common cause, accounting for 11.65% of medical admissions; followed by infection of the nervous system which was made up of cerebral malaria, pyogenic meningitis, and tetanus as the most common infections. All the other neurological diseases constituted less than 2% of medical admissions.[5] These studies showed a similar pattern; however, the difference in frequency rates in this study compared with the two studies may be related to the difference in the duration of the study periods. Our study compared well with a similar study carried out in Niger Delta, in which Neurologic diseases constituted 1.5% and 33.1% of hospital and medical admissions respectively with M: F ratio of 1.4:1 and mean age of 52.6 years. In his study the five topmost diseases were stroke (61.6%), meningitis and encephalitis (13.4%), tetanus (6.5%), spinal cord diseases (6.5%), and epilepsy (3.8%).[6] Analysis of outcome of these neurologic diseases showed that 753 (76.8%) were discharged, 219 (22.4%) died, and 8(0.8%) were discharged against medical advice or absconded. This findings compare with that of Chapp in Niger Delta.[6]

The duration of hospitalization of patients with neurologic diseases was usually long, ranging from less than 1 day observed mainly in patients who died soon after admission, to 96 days, with a mean of 25 days. Further analysis of stroke revealed that the peak age of admission was 57 to 65 years. This was in keeping with the finding of Chapp-Jumbo[6] and the previous findings that stroke is a disease of the elderly in Nigeria[7,8] and elsewhere[9] in contrast to earlier reports that stroke was more common below the age of 50 in the African,[9,10] a trend that may be related to the improving standard of living and longevity in our environment. However, some studies among the Caucasians showed a higher proportion of relatively younger Africans and Asians with stroke.[11] Twenty three percent of the cases of stroke died accounting for approximately 80% of all neurologic admissions; in Chapp-Jumbo review, the stroke mortality represented 65% of neurologic deaths, 18.8% medical deaths, and 2.37% hospital deaths. There was a slight male preponderance (M: F=3:2) as had been noted in the previous studies.[6–8] This is yet another evidence that stroke mortality is invariably on the high side also in the northern part of Nigeria. Odia and Wokoma[12] in their study reported that cerebrovascular disease was the most common cause of deaths in the medical wards of UPTH, accounting for 15.9% of medical deaths. In Ibadan, Nigeria, Adetuyibi *et al*.[13] also observed that stroke was the most common cause of neurologic deaths. The mean duration of hospitalization of stroke patients was 16.5 days and in Lagos Nigeria, Adegbite *et al*.[14] In this study cerebral infarct accounted for 69% of all stroke subtypes, this finding is in conformity with some other findings within and outside African continent.[15–17] Pathological patterns of stroke are different in various races because of variations in admission policy, diagnostic accuracy, age distribution, and related risk factors.[17] In Oxford, cerebral ischemia comprises >80% and ICH occurs in 10% to 15% of all strokes.[18,19] However, a high proportion (21% to 48%) of stroke type among American blacks and individuals of Chinese and Japanese ancestry is ICH.[20] The percentages are highly variable in different communities, and changing patterns have been reported in several communities including Nigeria.[7] In our study, ischemic stroke accounted for 69%, whereas hemorrhagic stroke accounted for 31%, the higher proportion of ischemic stroke is in keeping with some other studies[14,17,19,20] but at variance with the study of Ogun *et al*. in southwestern Nigerian in which ischemic accounted for 49% and hemorrhagic 45%,[21] this difference may be due to higher frequency of neuroimaging among stroke patients in our study. Generally, the real challenges of diagnosis and management of these neurologic diseases, in Nigeria, are inadequacy of neuroimaging facilities, lack of knowledge about stroke, and late presentation of the patients, to mention but few.

## Conclusion

In spite of the geographic uniqueness, the pattern of neurologic disorders warranting admission is not remarkably different from other parts of the country. Stroke appeared to be the most common neurologic admission and the most common cause of neurologic and medical death in Kano as observed in other regions of the country and a little over one-fifths of stroke patients die. Central nervous system infections mainly meningitis and tetanus are the next common cause of admission. Myelopathy, hypertensive encephalopathy, status epilepticus, Parkinson’s disease, cerebral tumors, cerebral malaria, and neuropathies are other causes of neurologic admissions in Kano. In view of these findings, the provision of a regional stroke unit with modern primary, secondary (including standard Intensive Care Units) and tertiary intervention facilities; the improvement of the sanitary conditions of the home and environment; the widespread use of immunizations against meningitis, tetanus cannot be overemphasized. These interventions will go a long way to reduce morbidity and mortality of stroke and neurologic infections.
